# Long-term functional outcome after tubular laparoscopic sigmoid resection for diverticular disease

**DOI:** 10.1007/s00384-023-04311-1

**Published:** 2023-01-16

**Authors:** Thaer S. A. Abdalla, Markus Zimmermann, Lena Weisheit, Michael Thomaschewski, Steffen Deichmann, Jan Nolde, Tobias Keck, Claudia Benecke

**Affiliations:** 1 Department of General Surgery, at the University Medical Center Schleswig Holstein, 23562 Lübeck, Germany; 2 Department of General Surgery, University Medical Center Schleswig Holstein, Campus Lübeck, Ratzeburger Alle 160, 23564 Lübeck, Germany

**Keywords:** Laparoscopic sigmoid resection, Incontinence, LARS, Diverticulitis

## Abstract

**Purpose:**

Sigmoid resection for diverticular disease is a frequent surgical procedure in the Western world. However, long-term bowel function after sigmoid resection has been poorly described in the literature. This study aims to assess the long-term bowel function after tubular sigmoid resection with preservation of inferior mesenteric artery (IMA) for diverticular disease.

**Methods:**

We retrospectively identified patients who underwent sigmoid resection for diverticular disease between 2002 and 2012 at a tertiary referral center in northern Germany. Using well-validated questionnaires, bowel function was assessed for fecal urgency, incontinence, and obstructed defecation. The presence of bowel dysfunction was compared to baseline characteristics and perioperative outcome.

**Results:**

Two hundred and thirty-eight patients with a mean age of 59.2 ± 10 years responded to our survey. The follow-up was conducted 117 ± 32 months after surgery. At follow-up, 44 patients (18.5%) had minor LARS (LARS 21–29) and 35 (15.1%) major LARS (LARS ≥ 30–42), 35 patients had moderate-severe incontinence (CCIS ≥ 7), and 2 patients (1%) had overt obstipation (CCOS ≥ 15). The multivariate analysis showed that female gender was the only prognostic factor for long-term incontinence (CCIS ≥ 7), and ASA score was the only preoperative prognostic factor for the presence of major LARS at follow-up.

**Conclusion:**

Sigmoid resection for diverticular disease can be associated with long-term bowel dysfunction, even with tubular dissection and preservation of IMA. These findings suggest intercolonic mechanisms of developing symptoms of bowel dysfunction after disruption of the colorectal continuity that are so far summarized as “sigmoidectomy syndrome.”

## Introduction

Diverticular disease has exponentially increased in the last century to become one of the most frequent gastrointestinal diseases in developed countries. Diverticulosis, which is defined by the presence of multiple asymptomatic diverticula in the colonic wall, affects up to 50% of patients older than 60 years [1]. Of those patients, 1–4% will develop acute diverticulitis which ranges from uncomplicated diverticulitis to diverticulitis with fulminant fecal peritonitis and will require medical or surgical intervention [2].

In the last decades, the treatment of diverticular disease has substantially changed. Historically, sigmoid resection was indicated after the second episode of diverticulitis or in complicated diverticular disease with concealed perforation. However, this has shifted into a more conservative approach, especially with increasing radiological interventions. Nowadays, indication for surgery is tailored in accordance with the patient’s complaints and objective criteria for stenosis and not just the number of episodes [3, 4]. Furthermore, the introduction of laparoscopy has shifted sigmoid resection from being an operation with a large incision and prolonged hospital stay into a minimally invasive procedure with minimal comorbidities [5].

Unlike for sphincter-sparing low anterior resection in rectal cancer, impairment of bowel function after non-oncologic laparoscopic sigmoidectomy has not been a matter of concern in clinical research. The topic was first described by Parks and Connel in 1970, who assessed 450 patients who underwent sigmoid resection. They were able to detect “mild abdominal symptoms” in almost a quarter of patients undergoing surgery at follow-up [6]. This was followed by other studies, which evaluated postoperative functional outcomes after sigmoidectomy. Moreaux and Vons [7] reported that 18% had “persistent abdominal pain/intestinal dysfunction” at follow-up. These studies attributed postoperative abdominal symptoms to concomitant irritable bowel syndrome and false operative indication by the presence of normal histology in the resected specimens [7, 8]. At first, Egger et al. [9] reported disturbed bowel function in 25% of patients after sigmoidectomy in patients with preoperative CT-proven diverticulitis. A major drawback in all named studies is that they did not use standardized validated questionnaires to evaluate patient symptoms postoperatively.

Bowel dysfunction can be divided into patients complaining of fecal urgency and incontinence, patients complaining of constipation and feelings of incomplete evacuation, or even sometimes, a combination of both. That is why several well-validated scales were introduced to discriminate patients’ symptoms and assess continence after colorectal surgery like the *Cleveland Clinic Incontinence Score* (CCIS) [10], the *Low Anterior Resection Syndrome Score* (LARS Score) [11] and the *Cleveland Clinic Constipation Score* (CCCS) [12]. Using these scales, bowel dysfunction after sigmoid resection for benign diseases has only been studied in a few studies [13, 14]. In contrast to sigmoid cancer, peripheral mesenteric dissection and preservation of the inferior mesenteric artery (IMA) are feasible during colon resection for diverticular disease. This technique has been shown to be associated with better bowel function after sigmoid resection [14]. Considering this information, our study aims to evaluate the influence of tubular sigmoid resection on long-term bowel function almost 10 years after surgery and to assess the perioperative parameters associated with bowel dysfunction at follow-up.

## Methods and materials

### Patient selection

Using a prospective database, we identified 638 consecutive patients who underwent laparoscopic sigmoid resection for diverticular disease between January 2002 and December 2011 at the University Medical Center Schleswig–Holstein (UKSH), Campus Lübeck. The indications for surgery were according to the previous surgical guidelines focusing on early operative intervention and were made before the introduction of current S3-German Guidelines for the treatment of diverticular disease [15].

We included patients who were aged ≥ 18 years and who underwent sigmoid resection with peripheral mesenteric resection and preservation of the inferior mesenteric artery. We included patients with both complicated and uncomplicated diverticular disease, who were operated on in recurrent disease. Patients who underwent a diverting loop ileostomy during the operation were included if they had a subsequent reversal and a postoperative follow-up questionnaire was collected after their ileostomy reversal.

To increase the response rate of the mail survey, a pre-mailing telephonic information campaign was conducted, where patients were introduced to the survey and its topics.

Of the identified 637 patients, 160 patients were lost to follow-up. Four hundred seventy-seven patients were mailed a standardized bowel function questionnaire. Of the 477 contacted patients, 238 patients responded to our questionnaire. The timeframe for accepted returns was set for 3 months from the day of sending.

The medical record for each patient in our study population was reviewed for patient demographics, medical comorbidities, preoperative medications, preoperative disease history, intraoperative findings, postoperative recovery, and complications. Moreover, the severity of the diverticulitis was assessed according to the Hansen–Stock classification. According to this classification, the severity is classified into three main clinical stages: stage 0 is asymptomatic diverticulosis, stage I is asymptomatic diverticulitis, stage IIa acute diverticulitis without abscess or perforation, stage IIb diverticulitis with abscess formation (concealed perforation), stage IIc diverticulitis with free perforation, stage III chronic diverticulitis [16]. This study was approved by the institutional review board and ethics committee at the University of Lübeck (AZ 15–302). All authors had access to the study data and reviewed and approved the final manuscript.

### Operative technique

All patients included underwent laparoscopic sigmoid resection. All operations had a lateral to medial approach with mesenteric dissection along the bowel wall preserving the inferior mesenteric artery. The level of distal and proximal colonic dissection was assessed intraoperatively depending on the intraoperative findings and extent of inflammation. All patients had stapled end-to-end anastomosis. The rare decision for a protective loop ileostomy was made intraoperatively.

### Survey of bowel function

We used previously validated questionnaires to assess different aspects of bowel function. The *Cleveland Clinic Constipation Score* (CCCS) was used for the assessment of constipation [12]. CCCS analyzes eight variables, which include frequency of bowel movements, painful evacuation, incomplete evacuation, abdominal pain, length of time per attempt, assistance for defecation, unsuccessful attempts for evacuation per 24 h, and duration of constipation. The items are scored from 0 to 4 for a maximum score of 30. A global score of more than 15 defines patients with constipation.

The *Cleveland Clinic Incontinence Score* (CCIS) [10] and the *Low Anterior Resection Syndrome Score* (LARS Score) [11] were used for the assessment of stool frequency and severity of fecal incontinence. CCIS is composed of five items and queries continence for solid and liquid stools, as well as gas, the usage of pads, and lifestyle alterations. The maximum score value is 20, representing complete incontinence. We grouped the patients into mild incontinence (1–6), moderate Incontinence (7–14), and severe incontinence (16–20). The LARS score was developed to evaluate the severity of low anterior resection syndrome (LARS) and includes control of flatus and liquid stool and bowel frequency, as well as clustering of stools and urgency. Patients are summarized into three groups according to their score: no LARS (0–20), minor LARS (21–29), and major LARS (30–42).

### Statistical analysis

Statistical analysis was done using *IBM SPSS ver. 26* (*Armonk*, *NY*, *USA*). The *t*-test was used for continuous variables. The *χ*^2^ test and Fischer’s test were employed for categorical variables. A *p*-value ≤ 0.05 was considered significant. Multivariate regression analysis was conducted to detect parameters associated with the presence of urgency and fecal incontinence at follow-up. The results were presented as hazard ratios with 95% CI. A *p-*value of less than 0.05 was considered statistically significant. The correlation between the LARS score, Cleveland Clinic Incontinence Score, and Cleveland Clinic Constipation Score with the time after surgery was assessed using Pearson’s coefficients.

## Results

### Patient characteristics

We retrospectively identified 637 patients, who underwent sigmoid resection for the diverticular disease at our institution between January 2002 and December 2011.

These patients were divided into two groups. The first group (group A) included patients who responded to the questionnaires and had available follow-up (*n *= 238). The second group (group B) included all other patients without available follow-up (*n *= 379). The mean follow-up time for responders was 117 ± 32 months.

To reduce possible sampling bias, both groups were compared regarding the baseline characteristics, perioperative parameters, and postoperative complications. Both groups were similar and differed mainly in their age (59.2 ± 10 years) in group A compared to (63.2 ± 12.8 years) group B (*p*-value < 0.001), operative time 152 ± 60 vs 163 ± 62 (*p *= 0.031) min, length of stay 7.7 ± 3 vs 9.2 ± 6 days (*p* < 0.001). However, both groups were similar concerning postoperative complications and disease severity according to the Hansen–Stock classification (Table 1).

**Table 1:** Comparison of baseline perioperative parameters between the responder and non-responder group

**Variables**	**Group A**	**Group B**	***p***
**Age**	59 ± 10	63 ± 12	< 0.001
**Sex**	106 (60.8%)	156 (55.6%)	0.2
**BMI**	26.7 ± 4	26.9 ± 5	0.707
**Intraoperative stoma**	7 (2.9%)	21 (4.5%)	0.376
**Conversion to open**	13 (3.3%)	5 (2.1%)	0.387
**Operative time**	152 ± 60	163 ± 62	0.031
**Hospital length of stay**	7.7 ± 3	9.2 ± 6	0.001
**Hansen and Stock**			
* Stage I*	9 (3.8%)	18 (4.5%)	0.401
* Stage IIa*	8 (3.3%)	13 (3.2%)	
* Stage IIb*	78 (32.6%)	136 (34.2%)	
* Stage III*	132 (55.2%)	195 (65.4%)	
* Missing*	9 (5.0%)	36 (9.0%)	
**ASA**			
* I*	27 (11.9%)	25 (6.6%)	< *0.001*
* II*	178 (78.4%)	269 (71.2%)	
* III*	22 (9.7%)	80 (21.2%)	
* IV*	0	4 (1.1%)	
* Missing*	11 (4.6%)	1 (0.3%)	
**Previous abdominal surgeries**	159 (66.5%)	275 (69.1%)	0.501
**Postoperative abscess**	4 (%)	1 (0.4%)	0.417
**Anastomotic leak**	4 (1.5%)	1 (0.4%)	0.202
**Postoperative sepsis**	4 (%)	1 (0.4%)	0.417
**Reoperation**	12 (5.0%)	30 (7.5%)	0.215
**Surgical site infection**	7 (1.8%)	4 (1.7%)	0.936
**Cardiac complications**	5 (1.3%)	1 (0.4%)	0.289
**Pulmonary complications**	10 (2.5%)	1 (0.4%)	0.049
**Renal complications**	1 (0.3%)	0	0.438

*P*-value indicates significance according to the *χ*^2^ test for categorical variables and *T*-test for continuous variables when patients with follow-up (group A) are compared with patients without follow-up (group B). Round parentheses indicate percentages

Group A (*n *= 238) included 131 (55%) female patients. The indication for surgery was uncomplicated diverticulitis in 8 (3.6%) patients, acute complicated diverticulitis type A in 8 (3.6%) patients, acute complicated diverticulitis type B in 78 (35.3%) patients, and chronic diverticulitis in 127 (57.5%) patients. Fifty-two (21.8%) patients had a BMI ≥ 30 kg/m^2^. Six patients received a protective ileostomy at the time of surgery and one patient received a protective ileostomy after anastomotic revision due to an anastomotic leak. All patients included had undergone stoma reversal at the time of follow-up. Twenty patients (8.4%) had severe postoperative complications (Clavien–Dindo ≥ 3). In 12 patients, a reoperation was performed; of those, four patients were due anastomotic leak.

### Functional outcome

#### Urgency and incontinence

Urgency and incontinence were evaluated with LARS score and CCIS in 238 patients. One hundred fifty-eight patients had no LARS, 44 had a minor LARS, and 35 had major LARS (Table 2). The mean LARS score was 15 ± 11. On univariate analysis, increased LARS score was associated with ASA score (*p *= 0.012) and the severity of the Hansen–Stock classification (*p *= 0.036) and tended to have an association with postoperative abscess formation (*p *= 0.060) (Table 3). The multivariate regression analysis showed that the advanced ASA score was an independent prognostic factor for major LARS at follow-up (HR 2.85, CI 95% 1.23–6.52, *p *= 0.013) (Table 4).

**Table 2:** Incidence of bowel dysfunction at long-term follow-up after laparoscopic sigmoid resection

**LARS**	**No dysfunction**	**Minor LARS**		**Major LARS**	
	158 (66.4%)	44 (18.5%)		35 (15.1%)	

**CCOS**	**No constipation**	**Mild constipation**		**Overt constipation**	
	229 (97%)	7 (2.9%)		2 (0.8%)	

**CCSI**	**No dysfunction**	**Mild**	**Moderate**		**Severe**
	66 (27.7%)	137 (57.6%)	30 (12.6%)		5 (2.1%)

**Table 3:** Univariate analysis of the influence of perioperative parameters on different aspects of bowel dysfunction

**Variables**	**LARS** ***p***	**CCIS** ***p***	**CCOS** ***p***
Sex (female)	0.357	0.005	0.082
Age ≥ 65	0.455	0.511	0.538
BMI ≥ 30 kg/m^2^	0.842	0.560	0.910
Hansen–Stock	0.036	0.072	0.161
ASA	0.012	0.474	0.269
Obstipation	0.305	0.180	0.097
Dolichosigma	0.539	0.044	0.023
Stenosis	0.586	0.646	0.251
Previous surgeries	0.731	0.313	0.060
Intraoperative conversion	0.631	0.793	0.779
Intraoperative stoma	0.457	0.150	0.515
Splenic flexure mobilization	0.795	0.896	0.934
Blood transfusion	0.481	0.168	0.205
Postoperative ICU	0.110	0.672	0.050
Clavien–Dindo ≥ 3	0.539	0.784	0.930
Anastomotic leak	0.776	0.864	0.912
Postoperative abscess	0.060	0.074	0.912

*P*-value indicates significance according to the *χ*^2^ test for categorical variables and *T*-test for continuous variables

**Table 4:** Multivariate analysis of variables affecting different types of bowel dysfunction

**Major LARS (30–42)**			
**Variables**	**HR**	**CI 95%**	*p***-value**
Age, ≥ 65 vs. < 65 y	0.45	0.19–1.10	0.078
Sex	1.49	0.70–3.19	0.298
ASA	2.85	1.24–6.52	0.013
Hansen–Stock	1.25	0.75–2.08	0.237

**Incontinece CCIS ≥ 7**			
**Variables**	**HR**	**CI 95%**	***p***-**value**
Age	0.86	0.39–1.91	0.714
Sex	3.11	1.34–7.26	0.008
Dolichosigma	2.48	0.86–7.18	0.092

*p* indicates significance according to binary regression analysis comparing the specified variables. HR indicates hazard ratio

On the other hand, according to CCIS, 66 patients had no bowel dysfunction at follow-up. One hundred thirty-seven patients had mild symptoms, 30 had moderate incontinence, and 5 patients had severe incontinence (Table 2). On univariate analysis, the severity of incontinence according to CCIS was associated with the female sex (*p* < 0.001) and dolichosigma (*p* = 0.04) (Table 3). The multivariate analysis regression showed that female sex was the only independent prognostic factor for the presence of moderate to severe incontinence (CCIS ≥ 7) at follow-up (HR 3.1, CI 95% 1.34–7.18, *p* = 0.008) (Table 4).

### Constipation

The CCOS was used to evaluate obstructive symptoms at follow-up. The mean CCOS score was 3.6 ± 3. Mild constipation (CCOS = 10–14) was present in 7 patients. Overt obstructive defecation (CCOS ≥ 15) was only present in two patients at follow-up. Due to the low number of patients with overt constipation, a multivariate regression analysis was not conducted.

## Discussion

This study evaluates long-term bowel function after non-oncologic laparoscopic sigmoid resection in 238 patients with diverticular disease. Here, we demonstrate that a proportion of patients who undergo laparoscopic sigmoid resection will suffer from long-term bowel dysfunction, even when performed with peripheral mesenteric dissection and preservation of the inferior mesenteric artery. In our study, bowel dysfunction is evaluated using the LARS score, Cleveland Clinic Incontinence Score, and Cleveland Clinic Obstipation Score almost 10 years after surgery, which provides enough time for the patients to recover from any reversible damage and for new bowel habits to settle.

Our results demonstrate that female gender is the only prognostic factor for long-term incontinence (CCIS ≥ 7) and ASA score is the only preoperative prognostic factor for the presence of major LARS at follow-up. Our data lack preoperative continence scores; however, these operations were carried out by certified colorectal surgeons or under their supervision. So that we can confidently assume that an anastomosis will not have been performed if the patients had preoperative incontinence or if there were overt concerns regarding postoperative incontinence. Therefore, we considered the presence of major LARS and severe incontinence at follow-up as new findings were not present at the time of surgery.

The term “sigmoidectomy syndrome” which represents urgency, fecal incontinence, and obstructed defecation after sigmoid resection was described by Levack et al. [13] who found these symptoms in one-fifth of the patients at a mean follow-up time of 50 months. Although, in their study, bowel function was assessed using different questionnaires. Nevertheless, our study reports similar results. In our cohort, 18.5% and 15.1% of the patients suffer from minor or major LARS, respectively, and 14.6% suffer from moderate-severe incontinence (CCIS ≥ 7) at follow-up. These results suggest the possible presence of permanent bowel dysfunction after sigmoid resection namely “sigmoidectomy syndrome.”

Continence and defecation are complex processes that result from an interplay between the anal sphincter, anorectal sensation, reservoir function, and rectal emptying. In 2021, an international panel of experts defined the most important factors, which lead to the development of LARS after pelvic surgery, which include (i) a decrease in reservoir function, (ii) autonomic denervation, (iii) afferent sensory loss in the rectum, (iv) anal sphincter injury, (v) pelvic radiation, and (vi) the use of diverting stoma [17]. However, for colonic resection in diverticular disease, it is seldom required to dissect beyond the peritoneal reflection. Thus, a high anastomosis is usually feasible, preserving the volume and anatomy of the rectum and preventing the denervation of the rectal tube. Furthermore, a peripheral mesenteric dissection and preservation of the IMA lead to the preservation of the sympathetic nerves which run along with the IMA, preserving sympathetic innervation of the rectal stump and its associated function [18]. For this reason, Masoni et al. [14] compared bowel function in patients undergoing laparoscopic sigmoidectomy with and without sectioning of the IMA and reported better functional outcomes 6 months after the intervention when preserving the IMA. The fact that bowel dysfunction is prevalent despite peripheral dissection and IMA preservation suggests that other factors contribute to “sigmoidectomy syndrome.”

Some studies explored the human colonic motoric function using high-resolution manometry and observed retrograde propagating cyclic motoric patterns, most commonly in the rectosigmoid region. It was postulated that these motoric patterns limit the untimely flow of stool into the rectum and so were called “the rectosigmoid brake” [19]. The presence of these contractions is significantly decreased after distal colonic resection, especially in patients with LARS [20]. These findings could explain the presence of urgency and incontinence after sigmoid resection.

Furthermore, in our cohort, the mean follow-up time is 9.8 ± 2.6 (min 5–max 15) years. The total scores of LARS, CCIS, and CCOS are not associated with the time since surgery, suggesting that new permanent bowel habits settle at an earlier time after surgery than our follow-up time frame (Table 5). These results complement the findings of Posabella et al. [21] who found that worse GIQLI scores were present in patients operated on earlier to 5 years compared to patients with longer follow-up times.

**Table 5:** Correlation of different bowel function scores (LARS, CCIS, CCOS) with time since operation (months)

**Variable**	**Months since operation**	
	**Correlation coefficient**	***p*-value**
LARS	0.127	0.099
CCIS	0.151	0.093
CCOS	0.118	0.069

*p*-value significant if *p* < 0.01 according to Pearson’s correlation

The main limitation of our study is the lack of preoperative functional data or comparative control group like age-related control group of patients without surgery due to the retrospective design of the study. Moreover, due to the long follow-up time, reliable data on anastomic height, extension of oral resection, or specimen length were not available. Consequently, the association of these factors with bowel function at follow-up could not be assessed and our analysis could not be adjusted for potential confounding factors. Nevertheless, we report a long-term evaluation of bowel function after sigmoid resection using a strict distal mesenteric dissection technique and well-validated questionnaires in a large number of patients. Our findings fortify the current evidence that “sigmoidectomy syndrome” can be a sequela of laparoscopic sigmoidectomy. Although we agree that central dissection and high tie of IMA are important prognosticators for bowel dysfunction, however, peripheral mesenteric dissection does not exclude long-term bowel dysfunction and should be highlighted during patient counseling for resection of benign sigmoid diseases.

## Conclusion

Sigmoid resection for diverticular disease as well as division of the colon continuity can be associated with long-term bowel dysfunction, even when performed using distant mesenteric dissection and preservation of IMA, which should be highlighted during patient counseling for operative therapy.

## Data Availability

The datasets used during the current study are available from the corresponding author on reasonable request.
